# Selective Hippocampal Subfield Atrophy Mediates Cognitive Decline in Cushing's Disease

**DOI:** 10.1002/brb3.71030

**Published:** 2025-10-29

**Authors:** Zhebin Feng, Tao Zhou, Xinyuan Yan, Kunyu He, Hailong Liu, Xiaoteng Yu, Rong Lu, Zhiguo Ma, Xinguang Yu, Yanyang Zhang

**Affiliations:** ^1^ Department of Neurosurgery the First Medical Center of Chinese PLA General Hospital Beijing China; ^2^ Department of Neurosurgery Chinese PLA 942 Hospital Yinchuan Ningxia China; ^3^ Department of Psychiatry University of Minnesota Medical School Minneapolis Minnesota USA; ^4^ Department of Neurosurgery Xiong'an Xuanwu Hospital Xiong'an New Area Hebei China; ^5^ Department of Radiotherapy Beijing Tiantan Hospital Capital Medical University Beijing China; ^6^ Department of Urology Peking University First Hospital, Institute of Urology, Peking University, National Urological Cancer Center Beijing China

**Keywords:** Cognitive, Cushing's disease, hypercortisolism, hippocampal subfields

## Abstract

**Background:**

Cushing's disease (CD) provides insight into how prolonged high cortisol exposure affects brain structure. While CD patients show cognitive and emotional symptoms linked to hippocampal function and detailed analysis of hippocampal subfield changes remains limited.

**Methods:**

The study included 91 patients with active CD and 53 matched healthy controls who underwent T1‐weighted magnetic resonance imaging and comprehensive neuropsychological assessment. We employed voxel‐based morphometry, automated segmentation, and shape analysis to evaluate gray matter volume, subfield volumes, and hippocampal morphology. Clinical correlations of hippocampal subfield volumes were also explored.

**Results:**

Compared to controls, CD patients showed decreased hippocampal gray matter volume, particularly in the body and tail regions. Specific subfields, including presubiculum‐body, subiculum‐body, CA4‐body, and granule cell layer, showed significant volume reductions. Shape analysis revealed corresponding surface alterations. Notably, left CA4‐body and left GC‐ML‐DG‐body volumes mediated the relationship between cortisol levels and cognitive performance.

**Conclusions:**

CD patients exhibit distinct patterns of hippocampal atrophy affecting specific subfields, with changes correlating to hormone levels and cognitive symptoms. These structural alterations may serve as potential biomarkers for CD and provide insight into the mechanisms underlying cognitive dysfunction in hypercortisolism.

## Graphical abstract

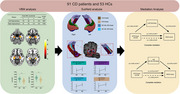

## 1 Introduction

Cushing's disease (CD) occurs when a pituitary tumor produces excess adrenocorticotrophic hormone (ACTH), leading to chronically high cortisol levels (Newell‐Price et al. 2006). This makes CD an ideal natural model for studying how prolonged cortisol exposure affects brain structure (van der Werff et al. 2015). CD patients experience both cognitive deficits (affecting memory (Piasecka et al. 2020; Resmini et al. 2012), verbal learning (Starkman et al. 2001), executive function, and attention (Piasecka et al. 2020)) and psychiatric symptoms (Na et al. 2020) (including depression, anxiety, and somatization). By studying CD patients, researchers can better understand how brain structural changes relate to these cognitive and psychiatric symptoms.

The hippocampus, a key component of the limbic system, controls learning, memory, emotion recognition, and attention (Aly and Turk‐Browne 2016; Sapolsky 2000). In CD patients, elevated cortisol levels appear to disrupt hippocampal function and structure, potentially explaining their cognitive and emotional symptoms. This disruption occurs through several mechanisms: reduced formation of new neurons (Anacker et al. 2013), decreased growth of support cells (astrocytes and oligodendrocytes) (McEwen et al. 2015), shortened nerve cell connections (Watanabe et al. 1992), and impaired memory processing (Piasecka et al. 2020). Additionally, high cortisol triggers inflammatory responses that can damage hippocampal tissue, as demonstrated in animal studies (Chesnokova et al. 2016; Vasic and Schmidt 2017; Zhao et al. 2019).

Previous research on hippocampal changes in CD has yielded mixed results. Early studies using manual measurements found significant hippocampal shrinkage (27%) that persisted even after treatment (Starkman et al. 1992; Starkman et al. 1999; Starkman et al. 2003). More recent studies using advanced imaging techniques (voxel‐based morphometry) confirmed reduced hippocampal volume and gray matter density (Burkhardt et al. 2015; Jiang et al. 2021; Li et al. 2022). However, some studies found no significant volume differences between CD patients and healthy controls (Frankowska et al. 2022; Resmini et al. 2012). These conflicting findings suggest the need for more detailed analysis of specific hippocampal regions, rather than examining only total volume.

The hippocampus is organized along a head‐body‐tail axis, with each section containing distinct subfields that can respond differently to disease. Examining only total hippocampal volume may obscure important changes in these specific regions. Recent advances in computer‐assisted analysis have enabled a more precise study of hippocampal structure. Using automated magnetic resonance imaging (MRI) analysis software developed by Iglesias et al. (2015), researchers can now reliably identify and measure 19 distinct subfields of the hippocampus. These include the parasubiculum, presubiculum (head and body), subiculum (head and body), cornu ammonia regions CA1, CA3, and CA4 (each in head and body), granule cell and molecular layers of the dentate gyrus (GC‐ML‐DG) (head and body), molecular layers (head and body), hippocampus‐amygdala‐transition‐area (HATA), fimbria, hippocampal tail, and hippocampal fissure. This detailed segmentation method has been validated as stable and effective, allowing researchers to examine subtle structural changes that might be missed in whole‐hippocampus analyses (Gao et al. 2020).

Studies across various neurological conditions have revealed specific patterns of hippocampal subfield changes. Insomnia severity correlates with reduced CA3/dentate gyrus volume (Neylan et al. 2010), while depression shows distinct changes in left CA1 volume that predict illness duration (Roddy et al. 2019). In temporal lobe epilepsy, volume loss occurs in CA, dentate gyrus, subiculum, and fimbria regions, matching patterns of neuronal loss (Schoene‐Bake et al. 2014). Parkinson's disease patients with depression show decreased CA3 volume (Györfi et al. 2017). Despite these findings in other conditions, research on hippocampal subfield changes in CD remains limited.

Our study aims to investigate hippocampal changes in CD patients with chronically elevated cortisol levels. We hypothesize that (1) CD patients will show reduced gray matter and subfield volumes compared to healthy controls, (2) these volume reductions will correspond to visible structural changes in hippocampal shape, and (3) these structural changes will correlate with both hormone levels and psychiatric symptoms, suggesting that high cortisol may cause psychiatric symptoms through its effects on specific hippocampal regions.

## 2 Materials and Methods

### 2.1 Participants

A total of 102 CD patients and 54 healthy controls (HCs) were recruited from the Department of Neurosurgery, The First Medical Center of Chinese PLA General Hospital, between May 2017 and October 2024. After excluding participants (including 11 CD patients and one healthy control) with low‐quality MRI images, the current study finally included 144 participants (91 CD patients and 53 HCs). Experienced endocrinologists diagnosed CD patients according to the latest clinical practice guidelines (Fleseriu et al. 2021): clinical characteristics (such as moon face, buffalo hump, and purple striae), high levels and abnormal circadian rhythms of relevant hormones (ACTH level at 8:00, reference range < 10.12 pmol/L; cortisol level at 8:00, reference range 198.7–797.5 nmol/L; 24‐h urinary free cortisol, reference range 98.0–500.1 nmol/24 h), special tests (negative results in dexamethasone suppression test and low dose dexamethasone suppression test, while positive result in high dose dexamethasone suppression test), inferior petrosal sinus sampling (inferior petrosal sinus to peripheral blood, ratio of ACTH level > 2), and histopathologic diagnosis (ACTH‐secreting pituitary adenoma). HCs were recruited from the local community and excluded any current or history of mental disorder by an experienced psychiatrist. All participants were right‐handed. This study adhered to the principles outlined in the Declaration of Helsinki and the ethical requirements from the local ethics committee (Ethics Committee Approval No. S2021‐677‐01). Before participating in the research, every patient provided written informed consent.

### 2.2 Clinical Data Acquisition

For CD patients, peripheral blood cortisol levels were measured at 0:00, 8:00, and 16:00; ACTH levels at 0:00, 8:00, and 16:00; and urinary‐free cortisol levels within 24 h. For HCs, peripheral blood's cortisol level at 8:00, ACTH level at 8:00, and urinary free cortisol level within 24 h were measured. A comprehensive neuropsychological assessment was also included in this study, such as the Self‐Rating Depression Scale (SDS), Self‐Rating Anxiety Scale (SAS), Montreal Cognitive Assessment (MoCA), and Cushing's Quality‐of‐Life (QOL) questionnaire (only for CD patients).

### 2.3 MRI Image Acquisition

High‐resolution T1‐weighted structural images were acquired using the GE Discovery MR 750w 3.0‐Tesla system (8‐channel head coil) at PLA General Hospital, Beijing. The parameters were shown as follow: repetition time = 6700 ms, echo time = 29 ms, flip angle = 7°, field of view = 256 × 256 mm^2^, voxel size = 1 × 1 × 1 mm^3^.

### 2.4 Preprocessing and Voxel‐Based Morphometry (VBM) Analysis

The image was converted to NIFTI format using MRIcron (version 1.0.20190902, https://www.nitrc.org/projects/mricron/), and image quality was checked manually. The structure image was preprocessed with the SPM 12 software (https://www.fil.ion.ucl.ac.uk/spm/software/) and the CAT 12 toolbox (http://www.neuro.uni‐jena.de/cat/) in the MATLAB environment. In short, the anterior commissure of the structural image should coincide with the origin (0,0,0) of the Montreal Neurological Institute (MNI) space. Then, segmentation (gray matter, white matter, and cerebrospinal fluid) (Ashburner and Friston 2000), space normalization (rigid and non‐rigid registration to MNI standard space), resampling (analysis with 1×1×1 mm voxel resolution and visualizations with 1.5×1.5×1.5 mm^3^), smoothing (8‐mm full‐width at half‐maximum), and total intracranial volume (TIV) calculation were conducted.

After data preprocessing, we assessed the difference in GMV between CD patients and HCs. A general linear model was constructed with sex, age, education year, and TIV as covariates. Apply absolute masking with a threshold of 0.2 (Ridgway et al. 2012). Next, a bilateral hippocampal mask based on the Anatomical Automated Labeling (AAL) template was applied to explore structural changes in the hippocampus. Using the false discovery rate (FWE) to correct for *p* < 0.05 at the cluster level to reduce Type 1 errors.

### 2.5 Segmentation of Hippocampal Subfields

The FreeSurfer (version 7.3.2, https://surfer.nmr.mgh.harvard.edu/) image analysis suite performed cortical reconstruction and volumetric segmentation (Fischl 2012; Sämann et al. 2022). Briefly, this processing includes motion correction and averaging of multiple volumetric T1‐weighted images, removal of non‐brain tissue, automated Talairach transformation, segmentation of the subcortical white matter and deep gray matter volumetric structures, intensity normalization, tessellation of the gray matter‐white matter boundary, automated topology correction, and surface deformation following intensity gradients to optimally place the gray/white and gray/cerebrospinal fluid borders at the location. Estimated TIV was calculated. The hippocampus was segmented into 19 subfields using Bayesian inference (Iglesias et al. 2015). The information on subfields was shown in Table S1. The results of automatic segmentations were visually inspected.

### 2.6 Hippocampal Shape Analysis

FSL‐FIRST (version 5.0.9, https://fsl.fmrib.ox.ac.uk/fsl/fslwiki/FIRST/) was used for automated segmentation and linear registration to the MNI152 space of the hippocampus (Patenaude et al. 2011). Image binarization and smoothing were conducted for each sample. Then FIRST modeled the boundary of all structures. The mesh parameterization (including vertices and edges) was performed using the spherical harmonic decomposition point distribution model (SPHARM‐PDM). Next, boundary correction was done to decide whether the boundary voxels should belong to the structure or not. Finally, a univariate test at each vertex was used to measure the difference in location between HCs and CD patients (Rahayel et al. 2019). The displacement vector of CD patients' vertices represented the deformation of the hippocampal surface relative to HCs.

### 2.7 Mediation Analysis

The bootstrapping method was employed to estimate the mediation effect (Preacher et al. 2007). Bootstrapping entails repeated random resampling from the existing data to empirically approximate the sampling distribution of a given statistic. This approximated distribution is then utilized to ascertain *p*‐values and construct confidence intervals (with a total of 5000 resamples). Furthermore, this methodology produces supplied confidence intervals (CIs) that undergo bias‐corrected and accelerated bootstrapping, ensuring greater accuracy and reliability (Preacher et al. 2007).

### 2.8 Statistical Analysis

Statistical analysis was performed using SPSS 25.0. Two‐sample *t*‐tests, *χ*2‐tests, and one‐way analyses of variance (ANOVA) were performed to assess GroupWise differences in demographics, clinical characteristics, and hippocampal subfield volumes. Mixed‐method ANOVA was conducted to evaluate the main effects and interactions of hemisphere and disease on hippocampal subfield volumes. Mauchly's test of sphericity was conducted to assess the assumption of sphericity for the within‐subjects effects. In the above analyses, sex, age, education year, and TIV were included as covariates. For the analysis of the 19 kinds of hippocampal subfield volume (each hemisphere), Bonferroni correction was applied as the multiple comparison correction to control Type 1 errors. Mediation analysis was performed to explore the relationship between hormone levels, hippocampal subfield volumes, and neuropsychological scale scores.

## 3 Results

### 3.1 Demographics

A total of 91 CD patients and 53 HCs were included in this study. No significant differences between CD patients and HCs were found for age, gender, or education years. The average duration of illness was 39.7 months in the CD patients. Compared with HCs, CD patients had higher levels of cortisol and ACTH, more severe anxiety and depression, pronounced cognitive impairment, and lower quality of life (Table 1).

**TABLE 1:** Demographic information of HCs and CD patients.

Characteristics	CD patients, *n* = 91	HCs, *n* = 53	*p*‐value
Gender, *n*_Male_/*n*_Female_, % female	9/82, 90.1%	3/50, 94.3%	0.378
Age, years, mean ± SD	38.95 ± 11.86	34.73 ± 10.05	0.131
Education years, mean ± SD	11.85 ± 4.17	11.92 ± 2.98	0.905
BMI, kg/m^2^, mean ± SD	26.57 ± 4.20	22.44 ± 3.18	**< 0.001**
Duration of illness, months, mean ± SD	39.70 ± 45.42		
QOL score, mean ± SD	35.90 ± 9.98		
SDS score, mean ± SD	43.20 ± 10.73	27.02 ± 4.41	**< 0.001**
SAS score, mean ± SD	42.27 ± 11.46	26.89±4.44	**< 0.001**
MoCA score, mean ± SD	22.97 ± 4.10	27.79 ± 1.73	**< 0.001**
cortisol level at 0:00, nmol/L, mean ± SD	574.70 ± 220.89		
cortisol level at 8:00, nmol/L, mean ± SD	726.31 ± 275.09	356.05 ± 108.40	**< 0.001**
cortisol level at 16:00, nmol/L, mean ± SD	656.42 ± 271.54		
ACTH level at 0:00, pmol/L, mean ± SD	15.36 ± 9.73		
ACTH level at 8:00, pmol/L, mean ± SD	20.18 ± 14.68	4.97 ± 3.07	**< 0.001**
ACTH level at 16:00, pmol/L, mean ± SD	19.40 ± 13.04		
UFC level within 24 h, nmol/24 h, mean ± SD	2,033.44 ± 1292.97	242.29 ± 117.51	**< 0.001**

*Note*: Two‐sample *t*‐test (for normal distribution variable) and *χ*2‐test (for classified variables) were performed to assess groupwise differences in demographics and clinical characteristics.

### 3.2 Voxelwise Statistical Analysis

**FIGURE 1:**
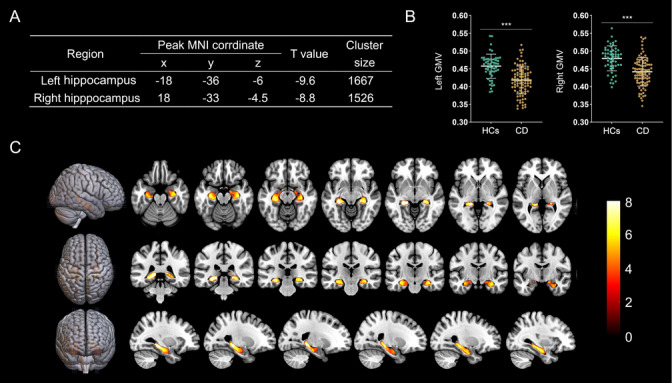
VBM analysis of the hippocampal region between HCs and CD patients. (**A)** The peak MNI coordinates of significant clusters. Compared with HCs, the GMV of the bilateral hippocampus of CD patients undergoes significant shrinkage (**B** and **C**). ***, *p* < 0.001, FWE corrected. Gender, age, education year, and TIV are included as covariates.

Compared to HCs, two clusters of reduced GMV in the CD patients in the left and right hippocampus were found (both right and left, *p* < 0.0001). The highest t score (9.6) was found within the cluster located in the left hippocampus, with peak MNI coordinates of ‐18 mm, ‐36 mm, and ‐6 mm. The cluster size was 1026 voxels. The highest t score in the right hippocampus was 8.8, with peak MNI coordinates of 18 mm, ‐33 mm, and ‐4.5 mm. The cluster size was 991 voxels (Figure 1). No cluster of increased GMV was found in the CD patients.

### 3.3 Hippocampal Volumetrics

Mixed‐method ANOVA revealed strong trends toward significance for the main effect for group for the whole hippocampus (*F* = 25.03, *p* < 0.001, *η* ^2^_p_ = 0.15) and hippocampal subfields (detailed in Supplemental Materials). There were no significant differences between the left and right hemispheres, nor the interaction effect of groups and hemispheres (Table S2). Mauchly's tests indicated no violation of sphericity (*p* > 0.05) in the above tests.

**FIGURE 2:**
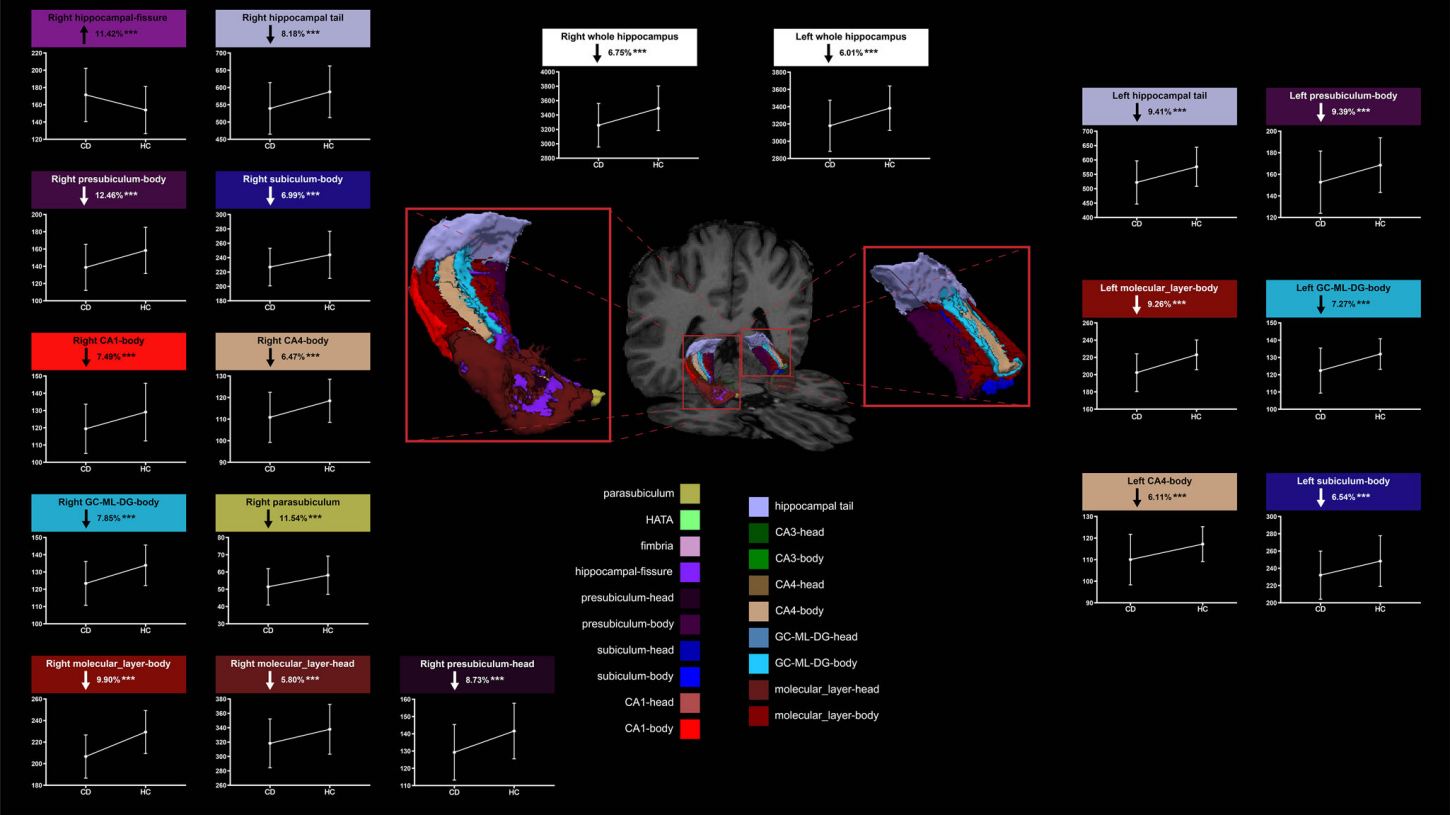
Hippocampal subfield analysis in CD patients versus controls. The hippocampus was segmented into 19 subfields using FreeSurfer, with significantly altered regions shown in opaque coloring while unchanged regions remain translucent. Bar graphs show volume differences as percentages. Affected regions demonstrated significant volume reductions (***p < 0.001, Bonferroni corrected), controlling for gender, age, education, and total intracranial volume. Detailed values are available in Table 2 and Table S1.

Further analysis using one‐way ANOVA, assessed with a Bonferroni‐corrected threshold of *p* < 0.0026 for 19 comparisons, confirmed bilateral hippocampal atrophy in CD patients (right *p* < 0.001 and left *p* < 0.001) (Table 2). Significant volume reductions were found in multiple regions: bilateral presubiculum‐body (right *p* < 0.001 and left *p* < 0.001), subiculum‐body (right *p* < 0.001 and left *p* < 0.001), CA4‐body (right *p* < 0.001 and left *p* < 0.001), GC‐ML‐DG‐body (right *p* < 0.001 and left *p* < 0.001), molecular_layer‐body (right *p* < 0.001 and left *p* < 0.001), hippocampal tail (right *p* < 0.001 and left *p* < 0.001), and right parasubiculum (*p* < 0.001), presubiculum‐head (*p* < 0.001), CA1‐body (*p* < 0.001), molecular‐layer‐head (*p* = 0.001), and hippocampal‐fissure (*p* = 0.001) had significant differences between HCs and CD patients (Figure 2). All these differences were highly significant (*p* < 0.001), suggesting widespread but specific patterns of hippocampal atrophy in CD patients.

**TABLE 2:** Volumetric between‐group differences for hippocampal subfields.

Subfields (mm^3^)	CD patients, *n* = 91	HCs, *n* = 53	*F*	*p*	*η* ^2^_p_
Left subfields					
Parasubiculum	57.65 ± 14.27	60.35 ± 9.73	1.072	0.302	0.008
Presubiculum‐head	133.69 ± 18.14	141.72 ± 15.38	8.110	0.005	0.056
Presubiculum‐body	152.76 ± 28.86	168.59 ± 25.30	15.163	**< 0.001**	0.099
Subiculum‐head	190.01 ± 27.33	200.30 ± 29.26	5.009	0.027	0.035
Subiculum‐body	232.08 ± 27.86	248.32 ± 29.35	15.543	**< 0.001**	0.101
CA1‐head	474.23 ± 56.20	492.58 ± 45.32	3.850	0.052	0.027
CA1‐body	111.73 ± 18.76	119.85 ± 16.23	6.586	0.011	0.046
CA3‐head	105.23 ± 16.15	105.37 ± 12.76	0.002	0.961	< 0.001
CA3‐body	78.89 ± 13.25	80.87 ± 11.11	1.161	0.283	0.008
CA4‐head	111.81 ± 12.83	116.39 ± 10.75	4.148	0.044	0.029
CA4‐body	110.04 ± 11.72	117.20 ± 8.07	19.067	**< 0.001**	0.121
GC‐ML‐DG‐head	134.56 ± 16.13	140.26 ± 12.86	4.134	0.044	0.029
GC‐ML‐DG‐body	122.39 ± 13.03	131.99 ± 8.85	28.227	**< 0.001**	0.170
Molecular_layer‐head	305.47 ± 32.57	319.46 ± 27.65	7.056	0.009	0.049
Molecular_layer‐body	202.37 ± 21.79	223.02 ± 17.21	39.958	**< 0.001**	0.225
HATA	51.26 ± 9.09	54.26 ± 7.01	3.254	0.073	0.023
Fimbria	83.01 ± 18.25	85.68 ± 11.97	0.540	0.464	0.004
Hippocampal_tail	522.08 ± 75.10	576.33 ± 67.98	20.533	**< 0.001**	0.130
Hippocampal‐fissure	155.34 ± 26.89	148.02 ± 29.45	2.055	0.154	0.015
Whole hippocampus	3,179.25 ± 296.72	3,382.53 ± 257.70	21.246	**< 0.001**	0.133
Right subfields					
Parasubiculum	51.44 ± 10.54	58.15 ± 11.09	13.594	**< 0.001**	0.090
Presubiculum‐head	129.26 ± 16.09	141.62 ± 16.13	21.212	**< 0.001**	0.133
Presubiculum‐body	138.67 ± 26.61	158.40 ± 26.80	21.549	**< 0.001**	0.135
Subiculum‐head	192.41 ± 25.42	207.43 ± 30.65	8.540	0.004	0.058
Subiculum‐body	226.94 ± 26.23	243.99 ± 32.81	16.009	**< 0.001**	0.104
CA1‐head	506.69 ± 62.29	533.26 ± 59.98	5.710	0.018	0.040
CA1‐body	119.42 ± 14.27	129.09 ± 16.68	13.611	**< 0.001**	0.090
CA3‐head	112.75 ± 16.58	111.79 ± 14.32	0.245	0.621	0.002
CA3‐body	85.90 ± 12.03	86.46 ± 12.61	0.189	0.664	0.001
CA4‐head	118.30 ± 13.42	123.64 ± 12.81	4.399	0.038	0.031
CA4‐body	110.85 ± 11.59	118.51 ± 10.00	20.668	**< 0.001**	0.130
GC‐ML‐DG‐head	142.54 ± 17.19	149.57 ± 15.39	4.979	0.027	0.035
GC‐ML‐DG‐body	123.38 ± 12.71	133.89 ± 11.75	29.315	**< 0.001**	0.175
Molecular_layer‐head	318.30 ± 33.98	337.89 ± 34.63	10.619	**0.001**	0.071
Molecular_layer‐body	206.67 ± 20.05	229.38 ± 19.92	53.045	**< 0.001**	0.278
HATA	51.72 ± 8.29	56.21 ± 7.85	9.063	0.003	0.062
Fimbria	83.06 ± 18.18	86.82 ± 17.53	0.661	0.418	0.005
Hippocampal_tail	539.55 ± 74.75	587.63 ± 74.88	19.349	**< 0.001**	0.123
Hippocampal‐fissure	171.50 ± 30.86	153.92 ± 27.32	11.265	**0.001**	0.075
Whole hippocampus	3,257.87 ± 302.23	3,850493.74 ± 310.61	25.496	**< 0.001**	0.156

*Note*: The table shows between‐group volumetric differences for individual subfields following analysis of covariance correcting for gender, age, education years, and estimated total intracranial volume. Bold indicated survived the Bonferroni multiple comparison correction. Bonferroni‐corrected significance threshold of each hemisphere: 0.0026.

*η* ^2^_p_ describes effect size (0.01 = low, 0.06 = moderate, 0.14 = large).

Abbreviations: HCs, health controls; CD patients, Cushing's disease patients.

### 3.4 Shape Analysis

**FIGURE 3:**
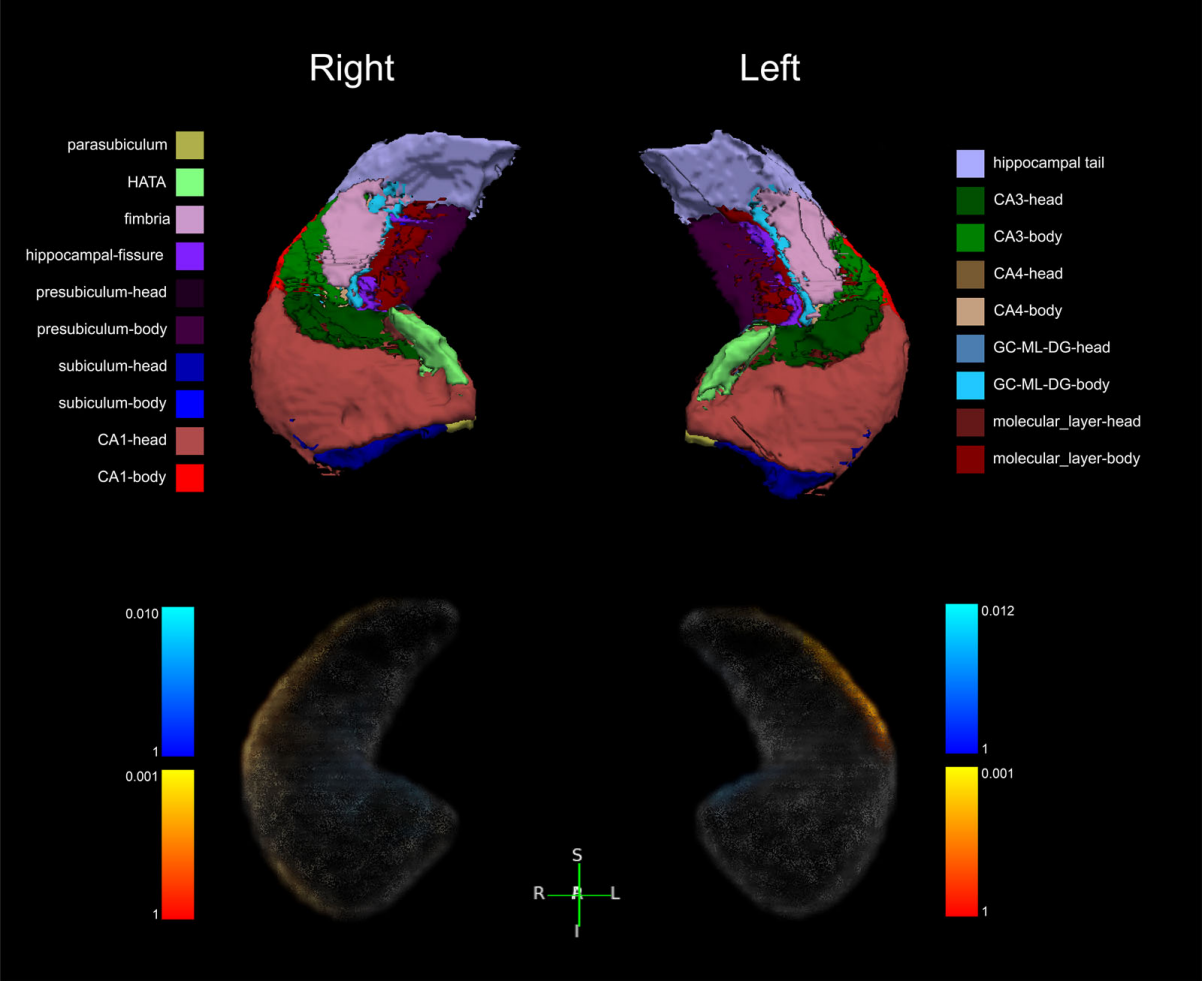
The morphological changes of the hippocampus of CD patients. The upper panel shows 19 subfields of the hippocampus, and the lower panel shows a 3D structure presentation of morphological changes in the hippocampus. Using the hippocampal surface of CD patients as the standard, the warm yellow region represents the prominent surface of HCs, while the blue region represents the depression of the hippocampal surface of HC. See Figure S1 for more information.

CD patients showed overall hippocampal shrinkage compared to healthy controls but with distinct patterns in each hemisphere. The left hippocampus primarily showed changes in its lateral body and tail regions, matching the areas of significant volume loss. The right hippocampus displayed more extensive changes, with depression across the lateral head, body, and tail regions. While the medial head of both hippocampi appeared enlarged, this likely reflects expanded cerebrospinal fluid spaces (widened fissures) rather than true hippocampal tissue expansion (Figure 3 and Figure S1).

### 3.5 Correlation Between Hippocampal Volume and Clinical Characteristics

**FIGURE 4:**
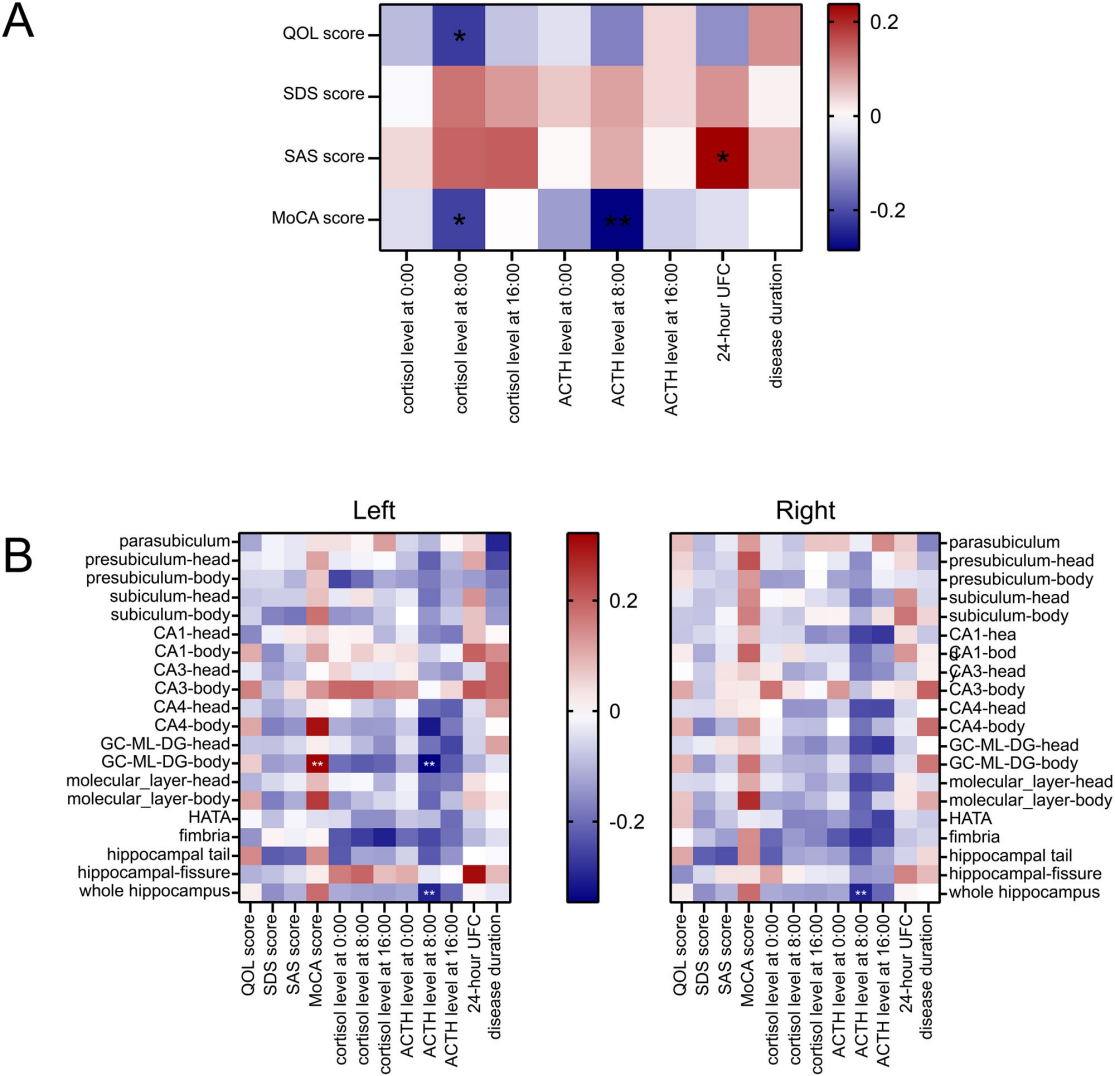
Partial correlation analysis of hormone levels, scale scores, and volumes of hippocampal subfields. (**A)** The partial correlation analysis of hormone levels and scale scores. (**B)** The partial correlation analysis of hormone levels, scale scores, and hippocampal subfield volumes. Gender, age, education year, and TIV were included as covariates. * indicates *p*‐value survives Bonferroni multiple comparisons correction. *, *p* < 0.05, **, *p* < 0.01.

For CD patients, partial correlation analyses showed significant correlations between the bilateral whole hippocampus and ACTH level at 8:00 (right *p* = 0.009 and left *p* = 0.005). The left GC‐ML‐DG‐body had a positive correlation with the MOCA score (*p* = 0.024) and a negative correlation with the ACTH level at 8:00 (*p* = 0.010). No significant correlation was found between right hippocampal subfields and hormone levels or scale scores (Figure 4).

**FIGURE 5:**
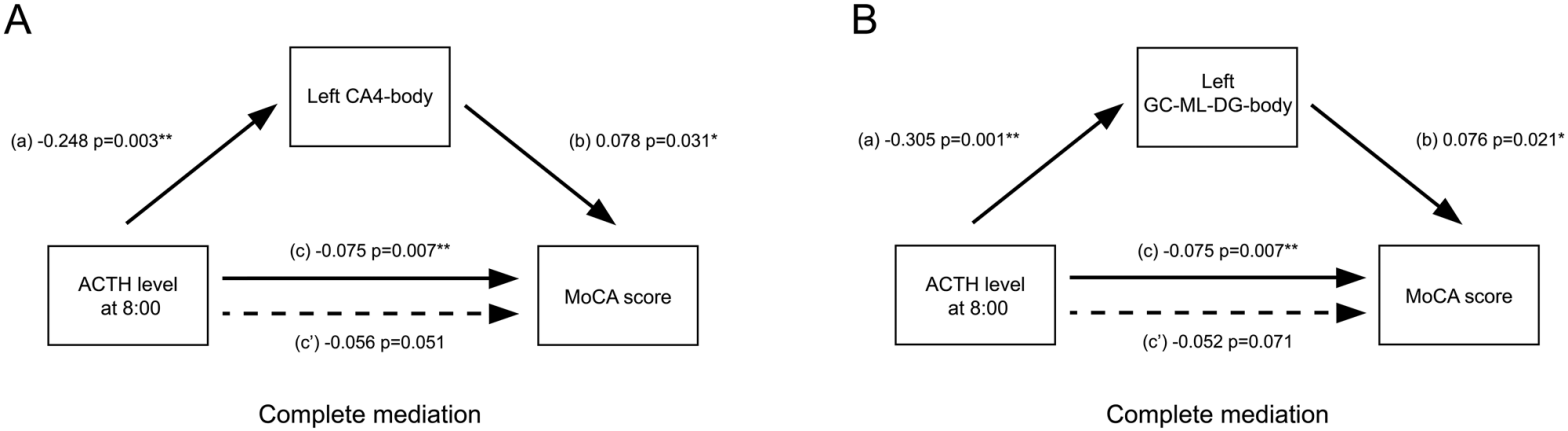
Mediation effect of hippocampal subfields between hormone levels and scale scores. The volume of the left CA4‐body (**A**) and left GC‐ML‐DG‐body (**B**) significantly completely mediated the relationship between hormone levels and scale scores. *, *p* < 0.05, **, *p* < 0.01.

Importantly, our mediation analysis revealed that higher morning ACTH levels led to reduced volume in two left hippocampal regions (GC‐ML‐DG‐body and CA4‐body), which in turn resulted in poorer cognitive performance. When controlling for these subfield volumes, the direct relationship between ACTH and cognitive performance disappeared, indicating these brain regions fully mediate the effect of high hormone levels on cognition (Figure 5).

## 4 Discussion

In this study of 91 CD patients and 53 healthy controls using T1‐weighted MRI, we found bilateral reductions in hippocampal gray matter volume, most pronounced in the body and caudal region. The right hippocampus showed more widespread atrophy than the left. Detailed subfield analysis revealed volume reductions in multiple regions, particularly in body and caudal subfields, with shape analysis confirming that structural depressions corresponded to areas of volume loss. Clinical correlations showed that total hippocampal volume was related to morning ACTH levels, while the left GC‐ML‐DG‐body volume correlated with both cognitive performance and ACTH levels. Most importantly, we found that two left hippocampal regions (CA4‐body and GC‐ML‐DG‐body) completely mediated the relationship between morning ACTH levels and cognitive performance, suggesting these structural changes represent the pathway through which elevated hormones affect cognition.

Our findings of reduced hippocampal volume align with several previous studies (Andela et al. 2015; Burkhardt et al. 2015; Chen et al. 2020; Jiang et al. 2021; Li et al. 2022; Starkman et al. 1992), though past research shows mixed results. While some studies report bilateral hippocampal atrophy in CD patients, others found no significant differences (Frankowska et al. 2022; Frimodt‐Møller et al. 2019; Resmini et al. 2012). Our detailed subfield analysis helps explain these contradictions: Volume reductions occur primarily in small subfields (parasubiculum, CA1‐body, CA4‐body, GC‐ML‐DG), which can be masked when measuring total hippocampal volume. Similar patterns of subfield‐specific atrophy have been observed in other conditions with elevated cortisol levels, supporting the selective vulnerability of these regions.

Using three complementary methods (VBM, subfield analysis, and shape analysis), we consistently found changes concentrated in the hippocampal body and tail regions. This selective vulnerability of posterior regions is particularly significant given their role in cognition and emotion. The body region contains critical pathways (perforant path, alveus, mossy fibers) (Modo et al. 2023) and shows stronger connectivity to the default mode network than anterior regions (Chang et al. 2021). Based on these findings, we hypothesize that high cortisol primarily affects posterior hippocampal regions, disrupting their connectivity and contributing to cognitive and emotional symptoms in CD patients.

Our correlation and mediation analyses revealed a clear pathway from hormone elevation to cognitive decline through specific hippocampal regions. Morning ACTH levels correlated with total hippocampal volume, while left GC‐ML‐DG‐body volume was linked to both ACTH levels and cognitive performance. Mediation analysis showed that volume changes in the left CA4‐body and GC‐ML‐DG‐body fully explain how elevated ACTH affects cognition. These findings align with known cellular mechanisms. The dentate gyrus (DG), particularly its granule cells, is highly sensitive to cortisol levels (Leuner and Gould 2010), while normal cortisol is necessary for cell survival, chronic elevation impairs neurogenesis and disrupts synaptic plasticity (Sloviter et al. 1989). Through mossy fibers and fornix connections, CA4/DG regions influence broader cognitive networks (Fitzsimmons et al. 2009; Modo et al. 2023). Similar patterns of CA4/DG volume reduction appear in other conditions with cognitive impairment (Doran et al. 2023; Foo et al. 2017). These results suggest targeted mechanisms for cognitive decline in CD and identify potential therapeutic targets for future drug development and neuromodulation strategies.

While both hemispheres showed changes, right hippocampal atrophy was more widespread. This asymmetry may reflect known functional differences between left and right hippocampi in verbal and spatial processing (Burgess et al. 2002; Li et al. 2022; Zhong et al. 2019), warranting future investigation of connectivity patterns.

Several key limitations warrant discussion. While our sample size was substantial, the cross‐sectional design prevents conclusions about temporal relationships and causality. Technical limitations also affected our imaging analysis. Our use of 3T T1‐weighted images, while standard, provides less detail than T2‐weighted or 7T imaging for visualizing hippocampal subfields, particularly the molecular layer and CA1 boundaries (Giuliano et al. 2017; Iglesias et al. 2015; Sämann et al. 2022). High‐field MRI with advanced segmentation techniques has demonstrated higher accuracy and revealed novel biomarkers in some diseases such as Alzheimer's disease and epilepsy (Wisse et al. 2016) (Adeyemi et al. 2025) (Pai et al. 2021). Future studies using higher‐resolution multimodal (combined T1‐ and T2‐weighted sequences) imaging could improve subfield segmentation accuracy. Quality control presented another challenge. FreeSurfer's hippocampal segmentation tool lacks standardized quality control procedures, relying primarily on visual inspection. To address this, we implemented CAT12 quality control and excluded images with quality ratings below 80% (Gaser et al. 2024). Additionally, although we visually inspected the automatic segmentation results, volume measurements of smaller structures (GC‐DG, CA4, molecular layer) may be less reliable (Iglesias et al. 2015), a common challenge in hippocampal subfield analysis due to their size and variable manual annotation standards. Despite these limitations, FreeSurfer's automated segmentation offers advantages over global hippocampal analysis (Pipitone et al. 2014), enabling the detection of subtle changes in CD patients while maintaining high stability and reducing manual segmentation bias.

## 5 Conclusion

In this comprehensive analysis of hippocampal structure in Cushing's disease, we demonstrated specific patterns of volume reduction and shape changes using multiple imaging methods (VBM, automated segmentation, and shape analysis). Key findings include decreased gray matter and subfield volumes in targeted regions, with corresponding shape alterations. Importantly, changes in specific hippocampal subfields correlated with cognitive impairment, suggesting these structural changes directly contribute to CD symptoms. These distinct patterns of hippocampal atrophy under prolonged cortisol exposure not only advance our understanding of CD's effects on brain structure but may also serve as potential biomarkers for the disease.

## Author Contributions

**Zhebin Feng**: writing–original draft, methodology, formal analysis, software, visualization. **Tao Zhou**: writing–original draft, data curation, validation. **Xinyuan Yan**: writing–review and editing, formal analysis, visualization. **Kunyu He**: writing–review and editing, investigation, software. **Hailong Liu**: writing–review and editing, resources, methodology. **Xiaoteng Yu**: writing–review and editing, software, methodology. **Rong Lu**: writing–review and editing, visualization, data curation. **Zhiguo Ma**: writing–review and editing, investigation, data curation. **Xinguang Yu**: writing–review and editing, resources, methodology. **Yanyang Zhang**: writing–review and editing, conceptualization, supervision, funding acquisition, project administration.

## Funding

This work was supported by the National Science and Technology Major Project of the Ministry of Science and Technology of China (No. 2022ZD0210100), the National Natural Science Foundation of China (No. 81871087 and No. 82001798), and the Young Talent Project of Chinese PLA General Hospital (No. 20230403).

## Ethics Statement

This study adhered to the principles outlined in the Declaration of Helsinki and the ethical requirements from the local ethics committee (Ethics Committee Approval No. S2021‐677‐01).

## Consent

Before participating in the research, every patient provided written informed consent.

## Conflicts of Interest

The authors declare no conflicts of interest.

## Peer Review

The peer review history for this article is available at https://publons.com/publon/10.1002/brb3.71030.

## Supporting information




**Supporting Table 1:‐S2**: brb371030‐sup‐0001‐tableS1‐S2.docx

**Supporting Fig. 1**: The morphological changes of the hippocampus in CD patients. Using the hippocampal surface of CD patients as the standard, the warm yellow region represents the prominent surface of HCs, while the blue region represents the depression of the hippocampal surface of HC. Axial, coronal, and sagittal images are presented in order.

**Supplementary Materials**: brb371030‐sup‐0003‐SuppMat.docx

## Data Availability

The data that support the findings of this study are available from the corresponding author upon reasonable request.
