# Supplementary material for: Selective Hippocampal Subfield Atrophy Mediates Cognitive Decline in Cushing's Disease
Source: Brain Behav. 2025 Oct 29;15(11):e71030. doi: 10.1002/brb3.71030 (PMC12571980; doi:10.1002/brb3.71030)
Supplement: Supplementary file 1 — Supporting Table 1:‐S2: brb371030‐sup‐0001‐tableS1‐S2.docx [file BRB3-15-e71030-s001.docx]

| Parasubiculum | The parasubiculum is Brodmann’s area 49. The parasubiculum is considered periallocortex (~5-6 layers) and lies on the lower bank of the hippocampal fissure. The parasubiculum is the medial-most of the subicular cortices, with the presubiculum laterally and entorhinal cortex medially. The parasubiculum is relatively small compared to the subiculum and presubiculum. |
| --- | --- |
| Presubiculum | The presubiculum is Brodmann’s area 27. The presubiculum is periallocortex and has distinct superficial layers with a heavily myelinated molecular layer. The presubiculum makes up a large portion of the territory on the lower bank of the hippocampal fissure in the human brain and extends posteriorly to the retrosplenial region. The presubiculum lies between the parasubiculum (medially) and the subiculum (laterally). The contrast in the presubiculum is heterogeneous, with light and dark contrast in its superficial layer (the lamina principalis externa), making it a particularly distinctive pattern in ex vivo MRI. The lamina principalis externa of the presubiculum ends at the subiculum. |
| Subiculum | The subiculum belongs to the allocortex group—three layered cortex with a molecular layer, a pyramidal layer, and a polymorphic layer. As the light and dark contrast of the presubiculum ends, the subiculum begins laterally. The boundary between presubiculum and subiculum is distinct because the subiculum has a well-defined pyramidal layer in ex vivo MRI. The pyramidal layer in the subiculum widens (compared to the presubiculum) and ramps up from a narrow wedge to a full-fledged allocortex. The molecular layer appears directly superior to the subiculum. It is between the presubiculum and subiculum that the cortex simplifies to a three-layered cortex. We did not segment the presubiculum. |
| CA1 | The subiculum transitions into CA1 laterally. CA1 displays light homogeneous contrast for the pyramidal layer, similar to the subiculum and other CA fields. The subiculum/CA1 boundary occurs approximately where the hippocampus turns upward (at 7 o’clock using the letter C as a representation for the hippocampus and radiologic convention for the right side). We labeled the hippocampal molecular layer separately from the CA1 pyramidal layer because we could distinguish the difference. CA1 continues until the top of the first hippocampal fold, where it meets CA2. CA1 dominates at the hippocampal head and lessens in the hippocampal body. The uncal (medial) portion of CA1 was included in the CA1 label. |
| CA3 | We combined subfields CA2 and CA3 due to lack of distinguishing contrast in MRI and variability among our labelers in preliminary experiments. We encountered great variability, particularly with the angle of the original CA2 label. CA2/3 showed a light intensity and homogeneous contrast as the pyramidal layer in CA1 but the pyramidal layer of CA2/3 appeared thinner than in CA1. The thickness change between CA1 and CA2/3 was a distinguishing feature to delineate these two subfields. When this change was gradual, the boundary was placed approximately in the center of the region of varying thickness. CA2/3 extended from the posterior half of the hippocampal head to the tail. CA2/3 was typically superior to the dentate gyrus but also weaved throughout hippocampal folds. Here, we also labeled the molecular layer in CA2/3 separately from the CA2/3 pyramidal layer. |
| CA4 | CA4 is also known as the hilar region of the dentate gyrus. Topographically, the CA4 subfield lies within the dentate gyrus. Thus, CA4 fills the interior of the GC-DG label. The limit between CA2/3 and CA4 is at the entrance of the hilus. The contrast of CA4 has a similar contrast to CA1-3 but a lighter contrast for the polymorphic layer. Thus, in ex vivo MRI with a FLASH sequence, it appears slightly darker intensity in the inner-most portion (i.e. the modified pyramidal area of CA4), but lighter outside of that (i.e. the polymorphic cell layer of the dentate gyrus). The ability to distinguish these particular strata depended on the brain quality and resolution. We included the polymorphic layer in our CA4 label. |
| GC-ML-DG | The dentate gyrus is another three-layered structure. The dentate gyrus consists of a molecular layer, a granule cell layer, and a polymorphic layer. The granule cell layer shows a bright white intensity with a FLASH sequence in ex vivo MRI, the intense contrast is likely due to the high packing density of the granule cells. The molecular layer of the dentate gyrus has a dark contrast and was included in the CA4 label because we could not always distinguish the stratum. The dentate gyrus begins about one-third to halfway through the hippocampal head from the rostral-most slices. The shape of the dentate gyrus varies depending on the cut plane. |
| HATA | The hippocampus-amygdala-transition-area (HATA) lies in the medial region of the hippocampus and is superior to the other subfields. The HATA shows a dark intensity compared to the CA subfields. Its base forms the medial and slightly dorsal border of the hippocampus, but this may depend on orientation. We consistently observed that the top of the hippocampal folds (i.e. the superior-most folds) were a tangential landmark to delineate the HATA inferior boundary. The inferior horn of the lateral ventricle borders the medial side of the HATA and the alveus borders the HATA laterally. |
| Fimbria | The fimbria is a white matter structure that extends from the alveus and eventually forms the fornix. The fimbria exits posteriorly to the mid-body level of the hippocampus and has the same dark intensity as the alveus. |
| molecular_layer | This label consists of two parts, the molecular layer for the subiculum and the molecular layer for CA fields. The molecular layer appears as a dark contrast that lies directly underneath the hippocampal fissure and above the subiculum. The molecular layer of the hippocampus continues as a dark contrast that forms between the CA regions and the GC-DG as well as the hippocampal fissure. The molecular layer follows the shape of the hippocampal folds. |
| Hippocampal-fissure | The hippocampal fissure opens up medially and extends laterally until it is a vestigial space between the molecular layers of the hippocampus and dentate gyrus. In ex vivo MRI, our scanning liquid (paraformaldehyde solution) fills the ventricle as cerebrospinal fluid would in the living brain. Air bubbles frequently appear as artifacts in this kind of imaging. |
| Hippocampal tail | The hippocampal tail has not been extensively studied in the neuroanatomical literature yet, so it is difficult to make reliable annotations in this region. Instead, we identified the first coronal slice (anterior to posterior) where the fornix is fully connected to the hippocampus and labeled the whole hippocampus with this “umbrella” label in the remaining slices (approximately 40). |

Table S1. Hippocampus subfield definitions

Referenced from: Iglesias, Juan Eugenio, et al. “A computational atlas of the hippocampal formation using ex vivo, ultra-high resolution MRI: Application to adaptive segmentation of in vivo MRI.” NeuroImage vol. 115 (2015): 117-37. doi:10.1016/j.neuroimage.2015.04.042.

| Subfields | Interaction  (Hemisphere × Group) | | | Hemisphere | | | Group | | |
| --- | --- | --- | --- | --- | --- | --- | --- | --- | --- |
|  | F | p-value | η^2^_p_ | F | p-value | η^2^_p_ | F | p-value | η^2^_p_ |
| Parasubiculum | 5.699 | 0.018 | 0.040 | 5.113 | 0.025 | 0.036 | 6.538 | 0.012 | 0.045 |
| Presubiculum-head | 4.342 | 0.039 | 0.031 | 3.515 | 0.063 | 0.025 | 15.808 | **< 0.001** | 0.103 |
| Presubiculum-body | 0.773 | 0.381 | 0.006 | 2.518 | 0.115 | 0.018 | 20.752 | **< 0.001** | 0.131 |
| Subiculum-head | 0.995 | 0.420 | 0.005 | 0.041 | 0.839 | 0.000 | 7.579 | 0.007 | 0.052 |
| Subiculum-body | 0.031 | 0.862 | < 0.001 | 1.320 | 0.253 | 0.009 | 17.889 | **< 0.001** | 0.115 |
| CA1-head | 1.386 | 0.241 | 0.010 | 0.766 | 0.383 | 0.006 | 5.241 | 0.024 | 0.037 |
| CA1-body | 0.477 | 0.491 | 0.003 | 0.303 | 0.583 | 0.002 | 11.552 | **0.001** | 0.077 |
| CA3-head | 0.343 | 0.559 | 0.002 | 0.925 | 0.338 | 0.007 | 0.092 | 0.762 | 0.001 |
| CA3-body | 0.518 | 0.473 | 0.004 | 0.096 | 0.757 | 0.001 | 0.757 | 0.386 | 0.005 |
| CA4-head | 0.034 | 0.853 | < 0.001 | 0.011 | 0.918 | 0.000 | 4.975 | 0.027 | 0.035 |
| CA4-body | 0.036 | 0.849 | < 0.001 | 1.968 | 0.163 | 0.014 | 23.468 | **< 0.001** | 0.145 |
| GC-ML-DG-head | 0.250 | 0.618 | 0.002 | < 0.001 | 0.999 | 0.000 | 5.171 | 0.025 | 0.036 |
| GC-ML-DG-body | 0.093 | 0.761 | 0.001 | 1.686 | 0.196 | 0.012 | 34.170 | **< 0.001** | 0.198 |
| Molecular_layer-head | 1.712 | 0.193 | 0.012 | 0.527 | 0.469 | 0.004 | 9.705 | **0.002** | 0.066 |
| Molecular_layer-body | 0.527 | 0.469 | 0.004 | 1.104 | 0.295 | 0.008 | 53.017 | **< 0.001** | 0.278 |
| HATA | 1.854 | 0.176 | 0.013 | 0.211 | 0.646 | 0.002 | 7.102 | 0.009 | 0.049 |
| Fimbria | 0.029 | 0.865 | < 0.001 | 1.647 | 0.201 | 0.012 | 0.738 | 0.392 | 0.005 |
| Hippocampal_tail | 0.056 | 0.813 | < 0.001 | 1.234 | 0.269 | 0.009 | 22.428 | **< 0.001** | 0.140 |
| Hippocampal-  fissure | 8.322 | 0.005 | 0.057 | 0.195 | 0.660 | 0.001 | 6.729 | 0.011 | 0.046 |
| Whole hippocampus | 1.433 | 0.233 | 0.010 | 2.877 | 0.092 | 0.020 | 25.031 | **< 0.001** | 0.154 |

Table S2. Hippocampus subfield results with the Mixed-method ANOVA model

The table shows between-group and between-hemisphere differences, as well as interaction between them for individual subfields following analysis of covariance correcting for gender, age, education years, and estimated total intracranial volume.

HCs, health controls; CD patients, Cushing’s disease patients.

Bold indicated survived the Bonferroni multiple comparison correction. Bonferroni-corrected significance threshold: 0.0026

η^2^_p_ describes effect size (.01 = low, .06 = moderate, .14 = large).
