# Supplementary figures and images for: Selective Hippocampal Subfield Atrophy Mediates Cognitive Decline in Cushing's Disease
Source: Brain Behav. 2025 Oct 29;15(11):e71030. doi: 10.1002/brb3.71030 (PMC12571980; doi:10.1002/brb3.71030)

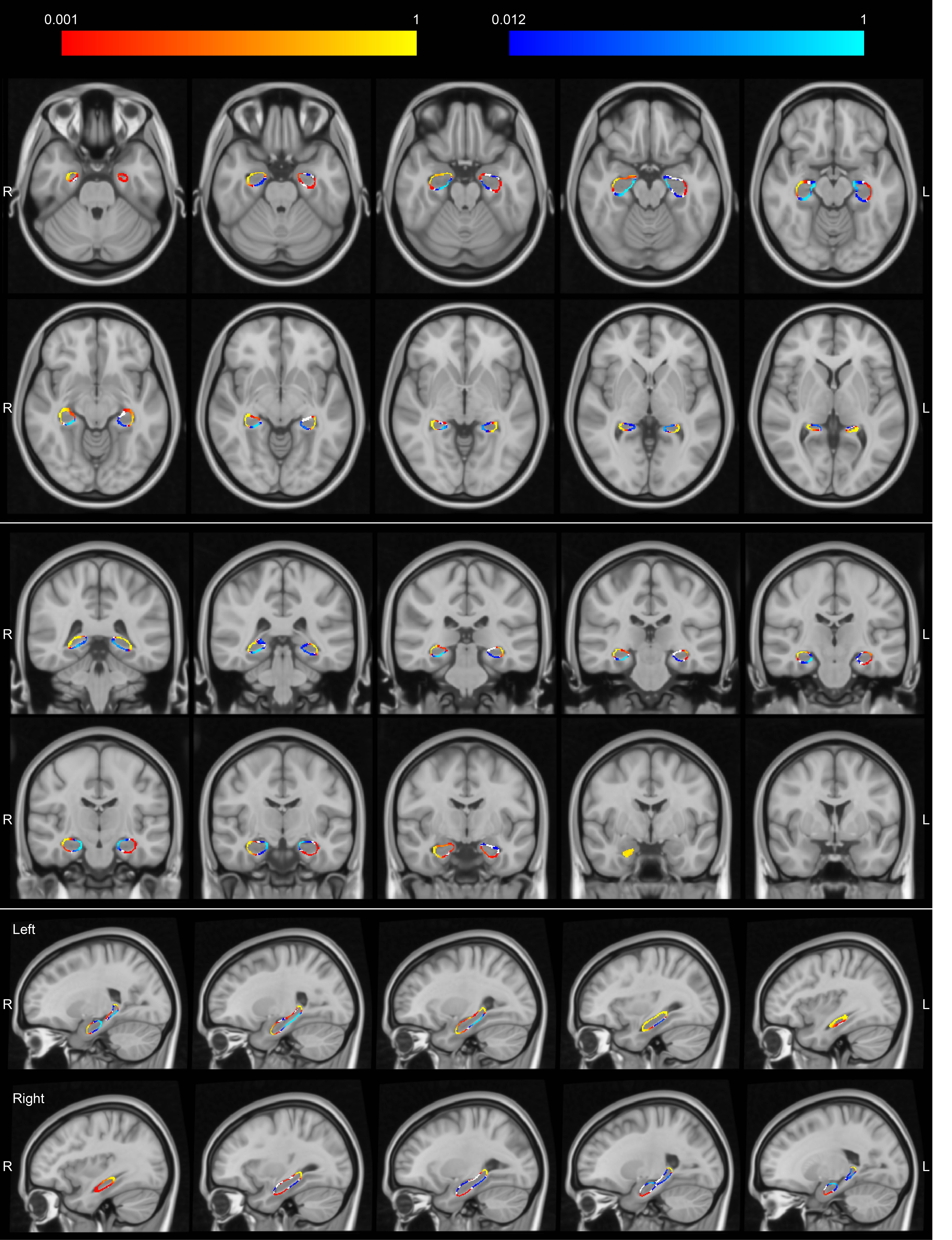

Supplement: Supplementary file 2 — Supporting Fig. 1: The morphological changes of the hippocampus in CD patients. Using the hippocampal surface of CD patients as the standard, the warm yellow region represents the prominent surface of HCs, while the blue region represents the depression of the hippocampal surface of HC. Axial, coronal, and sagittal images are presented in order. [file BRB3-15-e71030-s002.jpg]
