# Supplementary material for: Selective Hippocampal Subfield Atrophy Mediates Cognitive Decline in Cushing's Disease
Source: Brain Behav. 2025 Oct 29;15(11):e71030. doi: 10.1002/brb3.71030 (PMC12571980; doi:10.1002/brb3.71030)
Supplement: Supplementary file 3 — Supplementary Materials: brb371030‐sup‐0003‐SuppMat.docx [file BRB3-15-e71030-s003.docx]

**Supplemental materials**

1. Mixed-method ANOVA results of hippocampal volumetrics

Mixed-method ANOVA, assessed with a Bonferroni-corrected threshold of p < 0.0026 for 19 comparisons, revealed strong trends toward significance for the main effect for group for the whole hippocampus (F = 25.03, p < 0.001, η^2^_p_ = 0.15) and hippocampal subfields, including presubiculum-head (F = 15.81, p < 0.001, η^2^_p_ = 0.10), presubiculum-body (F = 20.75, p < 0.001, η^2^_p_ = 0.13), subiculum-body (F = 17.89, p < 0.001, η^2^_p_ = 0.12), CA1-body (F = 11.55, p = 0.001, η^2^_p_ = 0.08), CA4-body (F = 23.47, p < 0.001, η^2^_p_ = 0.15), GC-ML-DG-body (F = 34.17, p < 0.001, η^2^_p_ = 0.20), molecular_layer-head (F = 9.71, p = 0.002, η^2^_p_ = 0.07), molecular_layer-body (F = 53.02, p < 0.001, η^2^_p_ = 0.28), and hippocampal tail (F = 22.43, p < 0.001, η^2^_p_ = 0.14). There were no significant differences between the left and right hemispheres, neither the interaction effect of groups nor hemispheres (Table S2). Mauchly’s tests indicated no violation of sphericity (p > 0.05) in the above tests.

2.Correlation between Hippocampal Volume and Disease Duration

As for the disease duration, partial correlation analyses revealed no significant correlations between disease duration and whole hippocampal volumes, clinical scale scores, or any hippocampal subfield volumes after Bonferroni correction for multiple comparisons (corrected threshold p < 0.0026), controlling for sex, age, education, and total intracranial volume (TIV). While trends were observed for the left parasubiculum (r = -0.30, p = 0.005) and left presubiculum-head (r = -0.25, p = 0.021), these did not survive this rigorous correction.
